# Protocol for a core outcome set for pharmacological treatments in hospitalised patients with acute viral respiratory infections (COSAVRI)

**DOI:** 10.1371/journal.pone.0330288

**Published:** 2025-09-09

**Authors:** Declan Devane, Matthias Briel, Sanjay Bhagani, Nadine Boesten, Stephanie Buchholz, Estefania Callejas De Luca, Jeremie Guedj, Anastasia Koryakina, Kavita Kothari, Karine Lacombe, Patrick W. Mallon, Clément R. Massonnaud, Joanne O’Dwyer, Inge Christoffer Olsen, K. M. Saif-Ur-Rahman, Johannes M. Schwenke, James Thomas, Yazdan Yazdanpanah, Lina Zgaga, Julia Louw

**Affiliations:** 1 Center for Health Research Methods, School of Nursing and Midwifery, University of Galway, Galway, Ireland; 2 Health Research Board–Trials Methodology Research Network (HRB-TMRN), University of Galway, Galway, Ireland; 3 Evidence Synthesis Ireland and Cochrane Ireland, University of Galway, Galway, Ireland; 4 CLEAR Methods Center, Division of Clinical Epidemiology, Department of Clinical Research, University Hospital Basel, University of Basel, Basel, Switzerland; 5 Department of Infectious Diseases and HIV Medicine, Royal Free London NHS Trust and Division of Infection and Immunity, University College London, London, United Kingdom; 6 European Patients Forum, Brussels, Belgium; 7 Public Health Threats Department, European Medicines Agency, Amsterdam, Netherlands; 8 Division 32, Infectiology/Dermatology/Allergology, Federal Institute for Drugs and Medical Devices, Bonn, Germany; 9 Université Paris Cité, INSERM, IAME, Paris, France; 10 EPPI Centre, Social Research Institute, University College London, London, United Kingdom; 11 Sorbonne Université, INSERM IPLESP, Infectious Diseases Department, St Antoine Hospital, APHP, Paris, France; 12 Centre for Experimental Pathogen Host Research, School of Medicine, University College Dublin, Dublin, Ireland; 13 Département d’Épidémiologie, Biostatistique et Recherche Clinique, Hôpital Bichat, Université Paris Cité, Inserm, IAME, APHP, Paris, France; 14 Discipline of Pharmacy, School of Pharmacy and Medical Sciences, University of Galway, Galway, Ireland; 15 Department of Research Support for Clinical Trials, Oslo University Hospital, Oslo, Norway; 16 ANRS Emerging Infectious Disease, Inserm, Paris, France; 17 Department of Public Health and Primary Care, Institute of Population Health, Trinity College Dublin, University of Dublin, Dublin, Ireland; Public Library of Science, UNITED KINGDOM OF GREAT BRITAIN AND NORTHERN IRELAND

## Abstract

**Background:**

Acute viral respiratory infections (AVRIs) rank among the most common causes of hospitalisation worldwide, imposing significant healthcare burdens and driving the development of pharmacological treatments. However, inconsistent outcome reporting across clinical trials limits evidence synthesis and its translation into clinical practice. A core outcome set (COS) for pharmacological treatments in hospitalised adults with AVRIs is essential to standardise trial outcomes and improve research comparability.

**Objective:**

To develop an internationally agreed COS for pharmacological treatments in hospitalised adults ≥18 years with acute viral respiratory infections (COSAVRI) through stakeholder agreement.

**Methods:**

This protocol follows a four-stage development process in accordance with Core Outcome Set Handbook guidelines. Stage 1 comprises a rapid scoping review of randomised controlled trials (2015–2025) to systematically catalogue patient-relevant outcomes reported in pharmacological AVRI treatment studies. Semi-automated screening and data extraction will employ machine learning and large language models, with human verification. Stage 2 involves an online Real-Time Delphi survey with international stakeholders, including healthcare professionals, researchers, patients/caregivers, and policymakers, to prioritise identified outcomes using a 9-point scale. Stage 3 consists of structured online consensus meetings utilising anonymous electronic voting to finalise the COS. Stage 4 focuses on dissemination and implementation through academic publications, conferences, and stakeholder engagement.

**Expected outcomes:**

COSAVRI will provide a standardised minimum set of outcomes for measuring and reporting in future pharmacological trials involving hospitalised adults with AVRIs. This initiative will enhance evidence synthesis, reduce research waste, support regulatory decision-making, and improve pandemic preparedness by facilitating the rapid deployment of harmonised outcomes in trial protocols.

## Background

Acute viral respiratory infections (AVRIs) rank among the most prevalent illnesses globally and are a leading cause of hospital admissions and mortality. Each year, they cause seasonal surges that put pressure on healthcare systems across all regions [1–3]. In 2021 alone, respiratory infections resulted in an estimated 11.3 million deaths and approximately 350 million disability-adjusted life years (DALYs), with viral respiratory infections representing a substantial portion of this burden [4].

Viral respiratory infections in adults have shown significant epidemiological shifts, altering the transmission dynamics of various respiratory pathogens [5]. Of concern is the rise in hospitalisations linked to respiratory syncytial virus (RSV) among adults, particularly in older and immunocompromised populations, driven both by increased disease burden and improved diagnostic capabilities that have enhanced detection rates. This trend has prompted a renewed focus on prevention strategies, including vaccination for adults [6]. The introduction of molecular, multi-pathogen testing into routine clinical care has also led to an increase in diagnoses of respiratory viral infections and co-infections, notably influenza/SARS-CoV-2 combinations, which are associated with greater overall disease severity and resource utilisation in hospitalised adults [7]. Overall, the incidence of respiratory infections remains highest in low- and middle-income countries, where the burden is substantially higher than those in high-income settings, with viral respiratory infections contributing significantly to this burden [8].

Effective and approved pharmacological countermeasures, including virus and host-directed antiviral therapy, immunomodulators such as systemic corticosteroids, and other adjunctive therapies, are used in the management of AVRIs, particularly among medically vulnerable individuals, as they may help to reduce severity, duration, and both short- and long-term complications [9]. Despite the availability of various pharmacological treatments, high-quality clinical trials evaluating their efficacy in hospitalised adults with AVRIs remain limited, particularly for non-COVID viral respiratory infections. Among existing trials, methodological inconsistencies pose significant challenges, and determining the optimal outcomes for establishing the efficacy of new medical countermeasures is particularly problematic. Disease-specific regulatory guidance on the clinical evaluation of medicinal products already exists, for instance, for influenza (EMA/CHMP/VWP/457259/2014) and RSV (EMA/CHMP/257022/2017). The challenge for regulators lies not in the pathway itself, but in selecting clinically meaningful and reliably measurable outcomes, particularly when outcomes such as mortality and hospitalisation are both rare outside pandemic peaks and susceptible to regional care-standard bias. Thus, identifying alternative, patient-relevant outcomes depends on a consistent, evidence-based Core Outcome Set.

Contemporary pharmacological options span:

- Direct-acting antivirals (e.g., polymerase or protease inhibitors, neuraminidase inhibitors) that inhibit viral replication.
- Virus-neutralising and virus target-cell entry inhibiting monoclonal antibodies—these agents (e.g., palivizumab, nirsevimab) can prevent virus entry into cells and have shown prophylactic efficacy against, for example, RSV, and may also be used in combination with antiviral immunomodulators [10,11].
- Immune-based or host-directed therapies (e.g., interferons, systemic corticosteroids, JAK inhibitors, complement or inflammasome modulators) that modulate dysregulated inflammatory pathways; and
- Supportive or organ-protective medications that include anticoagulants to prevent or treat virus-associated thromboembolic events, vasopressors for circulatory support, and antioxidants to mitigate downstream complications.

Since SARS-CoV-2 emerged in 2019, global investments have accelerated the development and evaluation of a wide range of treatments, including small-molecule antivirals (e.g., remdesivir, molnupiravir, and nirmatrelvir/ritonavir), monoclonal antibodies, and host-directed anti-inflammatory/immunomodulatory therapies such as dexamethasone and baricitinib [12,13]. These developments not only highlight the therapeutic promise but also reveal methodological challenges in comparing diverse interventions across, and even within, individual pathogens, patient profiles, and health-care settings.

The lack of standardised outcome measures in trials, coupled with the inconsistent reporting of outcomes in AVRI trials, restricts the comparability of data across trials, the synthesis of data, and the translation of evidence [14]. This heterogeneity contributes to research waste and undermines patient care [15].

To address these inconsistencies, a Core Outcome Set (COS)—a minimum, standardised set of outcomes that all trials in a given field should measure and report—has been established [16]. A COS enhances the relevance, comparability, and applicability of research, ensuring that outcomes are meaningful to stakeholders, including patients and caregivers [17].

A critical consideration when developing any COS is whether trials should extend beyond traditional biomedical outcomes to encompass outcomes that are significant to patients and caregivers (e.g., quality of life, functional status post-discharge, treatment burden, and psychosocial impact). By engaging patients and caregivers in the consensus process, we ensure that these outcomes are considered alongside clinical measures.

Several existing COSs focus on COVID-19 [18–22]. However, outcomes reported in COVID-19 treatment trials (such as mortality, ventilation requirements, hospital discharge) are often generalisable across viral respiratory pathogens and will be included in our scoping review to inform the development of a pan-viral COS. Additional COS studies address COPD exacerbations [23], bronchiolitis [24], and acute respiratory failure [25]. However, none is specifically designed for pharmacological interventions in hospitalised adults with non-COVID-19 AVRIs, and existing COVID-19 COSs contain pathogen-specific elements that limit broader applicability, creating a need for a more generalisable approach across viral respiratory pathogens.

To address this gap, the Core Outcome Set for Pharmacological Treatments in Hospitalised Adults with Acute Viral Respiratory Infections (COSAVRI) will establish an internationally agreed-upon COS through collaboration among healthcare professionals, researchers, regulators, policymakers, and patient representatives.

## Methods

This protocol complies with the Core Outcome Set Handbook [16] and the COS-STAR reporting guideline [26]. It is registered with the COMET Initiative (https://www.comet-initiative.org/Studies/Details/3486) and on the Open Science Framework (https://osf.io/hev2f/files/osfstorage).

The COS development process comprises four sequential stages:

- Stage 1: A rapid scoping review to identify the outcomes reported in randomised controlled trials (RCTs) of pharmacological treatments for hospitalised adults with acute viral respiratory infections.
- Stage 2: An online, Real-Time Delphi survey involving international stakeholder groups—including patients, patient advocates/representatives and caregivers with lived experience of acute viral respiratory infections—to rate and prioritise Stage 1 outcomes.
- Stage 3: Online consensus meetings to finalise the core outcome set.
- Stage 4: Dissemination and implementation of the final COS.

## Stage 1: Rapid scoping review

### Objective

The aim of this rapid scoping review is to systematically catalogue all patient-relevant outcomes reported in RCTs assessing pharmacological treatments for adults hospitalised with a primary diagnosis of AVRIs, prioritising outcomes that directly reflect benefit or harm to patients (e.g., mortality, need for mechanical ventilation, time to hospital discharge) and excluding outcomes that cannot be addressed within the therapeutic trial framework (e.g., community viral transmission, population level economic metrics).

### Inclusion criteria

Inclusion criteria are given in Table 1.

**Table 1:** Scoping review inclusion criteria.

Domain	Criteria
**Study design**	RCT reports, including adaptive and platform trials, published between June 2015 and June 2025 (peer-reviewed or pre-print).
**Participants**	Adults (≥18 years) hospitalised primarily for laboratory-confirmed acute viral respiratory infection as the main reason for admission. Trials with mixed in-/out-patients are eligible if ≥80% (this threshold ensures the majority of participants reflect the target hospitalised population while allowing inclusion of studies with small outpatient components) of participants are hospitalised or hospital-specific results are reported separately.
**Interventions**	Any pharmacological therapy, or combination thereof, aimed at treating the viral infection or its immune-mediated sequelae (e.g., direct-acting antivirals, immunomodulators, monoclonal antibodies, corticosteroids, anticoagulants). Trials whose primary intent is prophylaxis or vaccination are excluded. Trials of supportive care interventions (e.g., oxygen therapy, mechanical ventilation protocols) without pharmacological intervention will be excluded.
**Comparator**	Placebo, standard of care, or active pharmacological comparator.
**Outcomes**	All reported outcomes irrespective of definition, measurement instrument or time-point. Particular attention will be paid to patient-centred outcomes to support meaningful PPI input later.

### Search strategy

We will conduct an electronic search of bibliographic databases. The search will be designed by an experienced information specialist and will cover Medline via Ovid, Embase via Elsevier, and Cochrane Central via Wiley. The search will be limited to randomised controlled trials (RCT) using a validated RCT filter. The publication date will be restricted to 2015 onwards to capture contemporary diagnostics and outcome measures. Given the rapid scoping review methodology, reference list screening will not be carried out to maintain feasibility within project timelines. No language restrictions will be imposed on the search; however, only English-language publications will be eligible for inclusion. We will therefore include an LLM prompt to exclude non-English records during screening.

Trial registries (WHO ICTRP portal and ClinicalTrials.gov) will not be systematically searched. This decision reflects methodological considerations specific to core outcome set development: (1) the primary objective is identifying the diversity and breadth of outcomes reported in the literature rather than achieving comprehensive study capture; (2) registry records typically provide limited detail on outcome definitions, measurement instruments, and timing compared to peer-reviewed publications, which are essential for meaningful outcome cataloguing; (3) substantial overlap exists between completed trials registered in these databases and those captured through comprehensive biomedical database searching; and (4) alignment with rapid scoping review principles that emphasise targeted, efficient evidence synthesis while maintaining methodological rigour. [27] The comprehensive search strategy across major biomedical databases with validated RCT filters provides sufficient coverage of contemporary outcome reporting practices necessary for COS development.

### Title- and abstract-level screening

Results will be imported into EPPI-Reviewer after deduplication in referencing management software. A machine-learning RCT classifier [28] will be used to identify potential RCTs, which will subsequently be screened against inclusion criteria using a large language model (LLM) to independently label records as “relevant”, “uncertain” or “irrelevant”. One reviewer (JL) will also audit LLM performance by independently screening the lesser of 10% or 500 records from each of the LLM “relevant” and “irrelevant” strata to validate the accuracy of the automated classification. We will implement prompts for each exclusion criterion individually and a composite ‘include’ prompt to allow for categorisation of all eligible studies. This will allow us to generate distinct exclusion codes for tracking and synthesis. Records not labelled “relevant” or ”irrelevant” by LLM will form the “uncertain” group and will undergo single human screening.

### Full-text eligibility assessment

All records that remain relevant after title or abstract screening will be retrieved in full text. Eligibility for full text will be assessed by one reviewer (JL) with support from a LLM, which will be used to assist in identifying relevant eligibility information. A second reviewer (JMS) will independently verify (i) every exclusion reason that involves uncertainty and (ii) a 20% random sample of inclusions; any disagreements will be resolved through discussion or, if necessary, by a third reviewer (KMSUR). All exclusions will be logged using CONSORT flow-diagram categories.

### Data extraction

Data extraction will use EPPI Reviewer’s GPT-enabled automated tool to capture study design, authors, publication year, journal, study country, patient population, targeted condition, diagnostic criteria, interventions tested, and reported outcomes (including measurement instruments and time points). Human reviewers (JL and JMS) will manually verify the accuracy of at least 200 randomly selected data items or 10% of all extracted items, whichever is greater. A sample of 200 provides a 95% confidence interval (CI) of ± 3 percentage points around a true discrepancy rate of 5% (n ≈ (1.96² × 0.05 × 0.95)/0.03² ≈ 203). If the discrepancy rate exceeds 5%, extraction prompts will be re-calibrated and re-audited. The a priori quality target is a discrepancy rate ≤ 5%.

After verification, we will calculate the point discrepancy rate and its 95% CI.

- Pass rule: if the upper CI limit is ≤ 8%, the automated extraction is accepted (this threshold provides approximately 80% power to declare success when the true error is 5%);
- Failure rule: if the upper CI exceeds 8% or the point estimate exceeds 5%, we will
  1. Increase the verified sample by 10% increments (or +200 items, whichever is smaller) and recalculate the CI;
  2. Retrain or modify the extraction model if the threshold remains unmet after three iterations;
  3. If the final discrepancy rate exceeds 5%, all remaining items will be subject to manual review.

A risk of bias assessment will not be performed, consistent with the PRISMA-ScR reporting guidance for scoping reviews [29].

### Data analysis and presentation

Data will be systematically organised using a cloud-hosted Microsoft Excel and AI-supported large language models (LLM). An up-to-date large language model (LLM)—selected at the time of extraction from the best-performing, publicly available models—will categorise outcomes into data-driven thematic domains. During the Steering Group review, established frameworks (e.g., COMET outcome taxonomy) may be consulted to identify gaps or overlaps, but the final domain labels will be chosen pragmatically, guided by the data and stakeholder judgement rather than by any single taxonomy.

The multi-disciplinary project Steering Group—including patient and public involvement (PPI) representatives—will review the automatically generated domains, merging or renaming them as appropriate, and approving the final domain structure. This Steering Group oversees the entire COSAVRI study and is distinct from the broader stakeholder groups that will participate in the Stage 2 Delphi survey. If substantial changes are made to the domain structure during the Steering Group review, the clustering will be iteratively refined to ensure outcomes are appropriately categorised within the finalised domains. Any major discrepancies between the initial automated clustering and the final Steering Group decisions will be documented and reported. The curated list of outcomes and their agreed domains will be exported for use in the Delphi survey and subsequent consensus meetings.

The rapid scoping review will be reported in accordance with the PRISMA-ScR reporting guidelines [29].

## Stage 2: Real-time Delphi survey

### Objective

To prioritise the outcomes identified in Stage 1 through an online Real-Time Delphi (RTD) survey.

### Participants

We aim to recruit at least 120 participants, comprising approximately 30 individuals from each of the four distinct stakeholder groups: healthcare professionals, researchers (including clinical trialists, virologists, epidemiologists, and health services researchers with expertise in viral respiratory infections and/or clinical trial methodology), patients and caregivers, regulators and policymakers. To ensure global representation, we will include participants from high-, middle-, and low-income countries across all stakeholder categories.

Our methodology requires the involvement of various international stakeholders, including those with direct experience of acute viral respiratory infections. This inclusive approach ensures that the outcomes prioritised for the COS genuinely reflect the values and needs of those most affected by these conditions. Recruitment of patients and caregivers will be backed by international patient advocacy networks and local patient groups, with support from patient representatives already involved in the project.

### Recruitment and engagement

We will utilise purposive sampling to ensure diverse representation of views and experiences. Participants will be recruited through professional networks, social media, patient advocacy groups, and healthcare institutions. Snowball sampling will be used to ensure adequate representation from low- and middle-income countries, focusing on maintaining geographical balance that reflects the global burden of acute viral respiratory infections while considering the European focus of the EU-PROACT funding framework. To encourage active participation, we will offer flexible response times, accessible digital platforms, and ongoing support throughout the process. To promote meaningful engagement of patients and caregivers, we will provide plain-language background materials, glossaries of key concepts, and optional briefing sessions prior to survey participation.

### Procedures

Delphi approaches in health sciences research have been extensively utilised to build consensus around key clinical topics [30]. The Delphi technique involves a panel of experts who provide iterative feedback to reach an agreement on a subject [31]. Typically, the traditional Delphi includes multiple rounds of surveys; however, this method can be resource-intensive and time-consuming due to its iterative nature [32]. We selected a real-time Delphi (RTD) approach because it maintains the methodological strengths of a classic multi-round Delphi while compressing timelines: participants can re-score items in real time after viewing anonymised, aggregated group feedback, and iterative rounds run automatically [33,34].

The outcomes identified in Stage 1 (rapid scoping review) will be shared with stakeholders through an online platform, Calibrum, which is specifically designed for RTD surveys. Participants will rate the importance of each outcome using a predefined 9-point Likert-type scale (1–3: not important; 4–6: important but not critical; 7–9: critical). After rating each outcome, participants will receive immediate (‘real-time’) feedback displaying the percentage of participants from each stakeholder group along with the overall participant rating for each point on the scale. To prevent early-respondent bias, aggregated feedback will remain concealed until a minimum of 30 participants (approximately 25% of the target sample and at least five from each stakeholder group) have completed their initial pass through the survey.

Participants will be encouraged to revisit the survey at their convenience to review group responses, enabling them to retain or adjust their ratings based on peer feedback [35]. Participants may propose up to five new outcomes. The study team will screen these suggestions daily for duplication and clarity; new items will be added to the live survey so that subsequent participants can rate them, and early respondents will be prompted by email to rate any items added later.

Calibrum automatically records the complete rating history for each participant (initial and revised scores), facilitating the analysis of score changes. All responses remain confidential; only the survey team can associate data with individuals. Participants view the rating distribution as percentages (no raw counts), and the order of outcomes within outcome domains is randomised for each user. The platform logs—but does not disclose—response counts, thereby limiting dominance and bandwagon effects.

At the close of the survey, outcomes will be classified as follows:

- Consensus-in: Outcomes rated 7–9 by at least 80% of respondents and rated 1–3 by fewer than 15% of respondents in at least three out of four stakeholder groups.
- Consensus-out: Outcomes rated 1–3 by at least 80% or more of respondents, and rated 7–9 by fewer than 15% of respondents in at least three out of four stakeholder groups.
- No consensus: All other outcomes will be discussed in consensus meetings.

If an unexpectedly large number of outcomes meet the ‘consensus in’ or ‘no consensus’ classification, potentially resulting in unmanageable consensus meeting agendas, the research team will review and may adjust the consensus criteria (e.g., requiring consensus in 4 out of 4 stakeholder groups) to ensure that the consensus meetings are productive and focused. Any such adjustments will be documented and reported transparently.

## Stage 3: Consensus meeting(s)

### Objective

The consensus meetings aim to secure agreement on the final COS through structured online gatherings with key international stakeholders who possess expertise in acute viral respiratory infections, as well as patients and caregivers with lived experience. These gatherings are designed to consider the diverse global stakeholders involved in respiratory infection care and management, prioritising broad international representation that includes individuals from low- and middle-income countries.

### Participants

All individuals who complete the Real-Time Delphi (Stage 2) will be invited to the consensus meetings; additional stakeholders may be purposefully recruited to ensure a balance of disciplines, geographies, and lived-experience perspectives. They will comprise a diverse and interdisciplinary group of international stakeholders: healthcare professionals (including but not limited to pulmonologists, infectious disease specialists, intensivists, clinical virologists, nurses, and allied healthcare professionals), researchers with expertise in clinical trials on pharmacological treatments, and patients along with their caregivers who have faced the challenges of acute viral respiratory infections, as well as regulators and policymakers.

### Procedures

We will conduct at least two online meetings (Zoom) to (i) accommodate global time zones and (ii) allow for reflection between sessions. Each meeting will include representatives from every stakeholder group. Pre-meeting materials will comprise (i) a concise agenda, (ii) a plain-language participant guide, (iii) a worksheet listing every candidate outcome with space for notes, (iv) a quick-view slide deck of Stage 2 ratings, and (v) printable “cut-out” outcome cards to assist participants in following the discussion; all will be circulated in advance in accessible formats. A 30-minute optional orientation call will be offered to new patient/caregiver members to guide them through Zoom functions and study terms. A designated technical support contact will be available for Zoom test calls and troubleshooting on the day.

We will use an anonymous, real-time electronic voting system during the meetings to gauge consensus on outcomes. Following discussions, participants will rate each outcome on a three-point scale: “Critical for inclusion,” “Important but not critical,” or “Not important.” *Consensus-IN* is achieved when ≥ 80% of all voters choose “Critical for inclusion”; any other result places the item *Consensus-OUT*. Outcomes initially falling just below the threshold (approximately 70–79% “Critical”) will be reopened for a second discussion and one final ballot; persistent non-consensus items will be excluded but recorded in the minutes. If fewer than 75% of Delphi participants attend the consensus meetings, we will conduct additional meetings or extend invitations to ensure adequate representation from all stakeholder groups. A designated plain-language facilitator will be available to clarify terminology for patients and caregivers. Screen breaks will occur approximately every 60 minutes, and participants are encouraged to mute their video and audio at any time. All deliberations will be chaired by an experienced moderator to ensure balanced input. Sessions will be recorded for audit and then deleted once the minutes are approved. Patient/caregiver attendees will receive an honourarium in line with INVOLVE guidance [36]. The set of outcomes reaching *Consensus-IN* will constitute the final COS for pharmacological treatments in hospitalised adults with acute viral respiratory infections.

## Stage 4: Dissemination and implementation strategy

Our dissemination plan will encompass academic, policy, and public-facing avenues. Results will be published as open access, presented at relevant infectious disease and trial methodology conferences, and shared with guideline developers and trial registry owners to encourage adoption in future studies. Targeted social media posts and webinars will extend our reach beyond academia.

To support interoperability, we will explore the mapping of each core outcome to existing data standards (e.g., CDISC or other open terminologies) where this can be achieved without compromising content. Any mapping undertaken will adhere to the FAIR principles—findable, accessible, interoperable, reusable [37]—to ensure that the COS can be easily incorporated into electronic case-report forms and shared datasets.

Materials intended for patients will be co-created with our PPI representatives.

### Ethical considerations

This study has received ethical approval from University of Galway Research Ethics Committee (Ref: 2025.05.031). All participants in the Real-Time Delphi survey (Stage 2) and consensus meetings (Stage 3) will provide informed written consent through online electronic or written consent forms prior to participation. Participation is entirely voluntary, and participants may withdraw at any time without providing a reason. All data will be collected and stored in accordance with applicable data protection regulations, including GDPR, where applicable. Patient and caregiver participants will receive appropriate support materials and briefings to ensure meaningful participation and will be compensated for their time. The study poses minimal risk to participants, as it involves only the completion of online surveys and attendance at virtual meetings.

### Study status and timeline

Study recruitment has not yet commenced. Participant recruitment is expected to be completed by February 2026, data collection will be completed by April 2026, and results are expected by June 2026.

## Discussion

This COS will enhance comparability and consistency across studies evaluating pharmacological treatments for acute viral respiratory infections in hospitalised adults, thereby harmonising outcome reporting in future trials and meta-analyses. By defining a minimum set of outcomes that all trials should measure, COSAVRI brings together the perspectives of key stakeholders, including healthcare professionals, researchers, regulators, policymakers, and patients and caregivers, ensuring that trial results remain both scientifically robust and clinically meaningful. Its widespread adoption is expected to streamline evidence synthesis, minimise research waste from heterogeneous or selectively reported outcomes, and provide a ready-to-use template for rapid trial deployment should another respiratory virus pandemic occur. In this way, COSAVRI represents both a methodological advance and a practical contribution to global pandemic preparedness.

**Disclaimer (required):** The views expressed in this article are the personal views of the authors and may not be understood or quoted as being made on behalf of or reflecting the position of the regulatory agency/agencies or organisations with which the authors are employed/affiliated.
